# Special Weapons and Tactics Occupational-Specific Physical Assessments and Fitness Measures

**DOI:** 10.3390/ijerph17218070

**Published:** 2020-11-02

**Authors:** Jessica Strader, Ben Schram, Shane Irving, Jeremy Robinson, Robin Orr

**Affiliations:** 1 Faculty of Health Sciences and Medicine, Bond University, 2 Promethean Way, Robina, QLD 4220, Australia; jessica.strader@student.bond.edu.au (J.S.); bschram@bond.edu.au (B.S.); 2 Tactical Research Unit, Bond University, 2 Promethean Way, Robina, QLD 4220, Australia; shane.irving@student.bond.edu.au; 3 Australian Federal Police, 47 Kings Avenue, Canberra 2600, ACT 2600, Australia; Jeremy.Robinson@afp.gov.au

**Keywords:** tactical, law enforcement, selection, fitness

## Abstract

Specialist tactical response police are required to frequently perform physically demanding tasks at high-risk capability levels, emphasizing the need for optimal physical fitness in this population. The aim of this study was to investigate the relationships between select measures of physical fitness and performance on an occupational-specific physical assessment (OSPA). A retrospective analysis on 18 male specialist police candidates (age = 32.1 ± 5.04 yrs; height = 183.72 ± 5.79 cm; body mass = 89.44 ± 8.56 kg; body mass index (BMI) = 26.45 ± 1.58 kg/m^2^) was conducted. Data were comprised of anthropometric measures, assorted fitness measures and OSPA performance scores. A stepwise linear regression determined the influence of measured fitness parameters on OSPA performance. A regression featuring both the 1 RM military shoulder press and grip strength of the non-dominant hand was the most significant predictor of performance (adjusted *r^2^* = 0.565, *p* = 0.001). A separate model, exclusively using the 1 RM military shoulder press additionally predicted OSPA performance (adjusted *r^2^* = 0.240, *p* = 0.023). These results emphasize the importance of optimal upper-limb muscular strength and its impact on key occupational tasks in specialist police candidates.

## 1. Introduction

Physical fitness is a necessary and crucial characteristic of tactical populations (law enforcement, military, and fire and rescue) due to the challenging and ever-changing nature of the job [1,2]. Personnel enlisted in these professions require the physical capability to successfully perform in a variety of high-risk and particularly dangerous situations [2,3]. While on duty, personnel may be required to sprint, crawl, vault over obstacles, pull or lift victims to safety and engage in life-threatening situations without notice [4,5,6].

Specialist tactical response law enforcement personnel, such as Special Weapons and Tactics (SWAT) officers, frequently resolve unique situations beyond that of general police [7]. These teams often perform high-risk tasks including tactical vehicle intercepts, warrant executions, resolve hostage situations, counter-terrorism response, water operations, counter-assault high-risk dignitary protection, rural operations and other unclassifiable, specialized tactical operations [1,3,7]. As a result, these officers frequently carry and use special weapons and tactics when threatened with arduous situations [3]. The specialized weapons and equipment carried by these officers can include semi and automatic rifles, hand guns, less lethal ammunitions, ballistic shields, body armour and breaching equipment [3], which can cumulatively weigh from 23 kg [8] up to in excess of 40 kg [9]. These loads are notably heavier than the loads of general duties law enforcement which have been reported to be approximately 10 kg [10]. It is the ability of a SWAT officer to perform advanced technical skills and tactics at higher intensities, while wearing these heavy loads, that constitutes the reason these officers are typically called on to handle the most dangerous jobs [5]. These physically demanding task requirements emphasize the need for excellent physical capacity in the specialist tactical population and can, at times, be the determinant between mission success and failure [2,6].

Research into the physical fitness of these specialist tactical response police suggests that they score higher across multiple fitness domains when compared to non-specialist tactical personnel and the general population in measures including strength, muscular endurance, flexibility, and aerobic fitness [7,11]. As an example, a critical review by Maupin et al. [11] found that elite tactical units (ETUs), inclusive of SWAT and Special Forces, score higher in push-ups, sit-ups, sit and reach assessments and have a higher relative VO_2 max_ when compared to general police and military units. Likewise, Pryor et al. [7] found that SWAT operators ranked high in tests of muscular strength, with individuals scoring in the 90th percentile for leg press strength and in the 85th percentile for bench press.

It has been acknowledged that meeting physical fitness standards is a critical aspect of preparedness in tactical populations, especially in specialist units [2]. To ensure the readiness of this population, it is common practice that specialist police candidates pass a multitude of assessments, including both fitness measures (e.g., vertical jump, push-ups, pull-ups, and 1 RM strength measures) and job-specific skill assessments (e.g., body drags, fence climbs, and obstacle courses) [1]. Past research has demonstrated the predictive abilities of select fitness measures on task performance. For example, Lockie et al. [1] found a mild-to-moderate correlation (range *r* = −0.127–0.574) and predictive relationship (*r^2^* range = 0.217–0.500) between fitness measures (e.g., push-ups, sit-ups, pull-ups, mountain climbers and aerobic fitness measures) and job-specific skill performance (e.g., 99 yard obstacle course run, chain link fence climb, solid wall fence climb, and 500 yard run) in a population of general duties police. However, how fitness measures relate to the more complex skills of specialist police while encumbered by their physical loads is yet to be investigated. Thus, the aim of this study was to investigate relationships between select measures of physical fitness and performance on a custom-designed occupational-specific physical assessment (OSPA) in specialist tactical response police candidates. This assessment was designed by the law enforcement agency to be reflective of the occupational requirements of specialist police, which could be used as an entry standard, a return to work assessment, or an annual work readiness assessment. It is hypothesized that, given the nature of the OPSA, anaerobic fitness measures will be highly related to performance in this assessment.

## 2. Materials and Methods

### 2.1. Participants

This study used a retrospective cohort design. Retrospective data, in a non-identifiable form, were provided for 18 male specialist tactical response police candidates (age = 32.1 ± 5.04 years; height = 183.72 ± 5.79 cm; body mass = 89.44 ± 8.56 kg; body mass index (BMI) = 26.45 ± 1.58 kg/m^2^) participating in barrier testing for service in an Australian specialist police force. Gatekeeper approvals were provided by the Australian law enforcement agency where the study took place. Prior to study commencement, the Bond University Human Research Ethics Committee (#15412) approved the use of this protocol.

### 2.2. Protocol

Data collected included demographic information (age), anthropometric measures (height, body mass, BMI, and loaded BMI), fitness measures (Illinois agility test, 1 RM bench press, 1 RM back squat, 1 RM military shoulder press, 1 RM hexagonal bar deadlift, grip strength, loaded pull-ups, 7 stage sit-up, push-ups, progressive shuttle run test and 1.2 km run) and a specially designed task performance measure (OSPA). The outcome measures were those commonly employed within this population as a tool for entry onto selection courses and as a measure of performance [12,13,14]. These tests were those chosen by the agency’s strength and condition coach and outside the control of the researchers.

Age, height, and body mass: Age (years) and height (cm) measurements were self-reported by selection course candidates. Self-reported height has been used previously in tactical populations and has been shown to be reliable [15]. The weight of the candidate was measured on the barrier test day using standard procedures [15] on a scale (Tanita BC82Fitplus, IL, USA).

BMI and LBMI: Body mass index (BMI) was calculated based on candidates’ self-reported height and weight measurements and using standardized procedures (BMI = weight (kg)/[height (m)]^2^) [16]. Loaded body mass index (LBMI), a new measure, was further calculated using the standardized BMI formula, with weight adjusted to combine the candidates usual body weight with the amount of load carried (LBMI = loaded weight (kg)/[height (m)]^2^) for the loaded tasks.

Illinois agility test: The Illinois agility test was administered using a standardized approach as described in previous literature [17]. The test was conducted on a flat, non-slip surface with eight cones arranged in a 9.14-by-9.14 metre square with four cones placed to mark the boarders of the square and another four cones aligned halfway through the agility course (4.57 m) so that the candidate could weave through the markers. When instructed, the candidate accelerated to the top corner cone, quickly turned and accelerated back towards the middle set of cones to weave up and back around the cones. Once weaving up and back through the four cones, the candidate accelerated to the furthest corner cone, again making a quick turn around and sprinting back to the finish. Candidates were familiar with the test and were allowed one attempt. Time was recorded and measured to the nearest 0.01 s using a handheld timer (Hart Sports, Zillmere, Australia).

1 RM strength protocol: Muscular strength is commonly documented as a measure of a repetition maximum (RM). A one repetition maximum (1 RM) load refers to the maximum amount of weight a person can lift in a single voluntary effort with proper technique [18,19]. Although a 1 RM lift is recognized to be the most accurate method to determine strength, it can often be time consuming. Alternative methods are widely used and include deriving a 1 RM from multiple-repetition sets (e.g., 3 RM) [18].

All candidates were familiar with 1 RM testing protocols having to record personal 1 RM data, derived from a 3RM lift, in a training diary and submit their results to the specialist unit’s head strength and conditioning coach as part of their pre-selection preparation. Each candidate converted their 3 RM to a 1 RM value for the (1 RM = weight × reps × 0.0333 + weight) bench press, back squat, military shoulder press and deadlift. As all candidates were required to track their results, they were familiar with this testing and protocol. Candidates completed the 1 RM testing in pairs in their own time under the supervision of the unit’s strength and conditioning coach using the Jim Wendler formula (1 RM = weight × reps × 0.0333 + weight) to convert their 3 RM to a 1 RM estimate. The final training cycle concluded two weeks prior to the OPSA testing. Standard protocols for these measures are described below [19].

1 RM bench press: Candidates completed the bench press using a 20 kg Pendlay brand barbell (MDUSA, SC, USA), Garage gym brand bumper weight plates (LifeFitness, Rosemont, IL, USA) and Hammer Strength bench support (LifeFitness, Rosemont, IL, USA). Standard bench press protocol required candidates to lay flat on their back, feet flat on the ground, with scapulae and buttocks in contact with the bench. The barbell was grasped at slightly wider than shoulder width to allow for a 90 degree angle when the bar was at the lowest point. Candidates were required to lower the weight in a slow, controlled manner until the barbell just touched the chest then pushed back up to the starting position (1 repetition).

1 RM back squat: Candidates completed the back squat using a 20 kg Pendlay brand barbell, Garage gym brand bumper weight plates and Hammer Strength Power Rack. The back squat protocol required candidates to step under the barbell located in the supporting arms on the power rack. Again, the barbell was grasped at slightly wider than shoulder width or was altered based on mobility of the individual. The candidate was required to position the barbell on their upper back, just above the trapezius. Standard foot width required feet to be just wider than hip-width apart. When ready, the candidate removed the weighted barbell from the rack supports, took two steps back then performed the eccentric movement of the squat, flexing at both knees and hips. Appropriate depth of the squat was determined when the central line of the femur was parallel to the ground. When successful depth was achieved the candidate extended the hip and knee joints and returned to the starting position (1 repetition).

1 RM military shoulder press: Candidates completed a military shoulder press using a Pendlay brand barbell, Garage gym brand weight plates and Hammer Strength Power Rack. Standard shoulder press protocol required candidates to step under the barbell located in the supporting arms on the squat rack. The shoulder press was conducted using a pronated grip (back of hands facing towards candidate) with a grip width wider than shoulder width to allow for 90 degrees of flexion at the elbow. Candidates were required to place the barbell over the clavicles, take two steps back and position their feet to be hip-width apart with a slight bend in their knees. When ready, candidates lifted the bar overhead to fully extend the elbows then eccentrically lowered the bar back down to the chest (1 repetition).

1 RM hexagonal bar deadlift (HBD): Candidates completed a deadlift using an Australian Barbell Company hexagonal deadlift bar (weighing 24 kg) (Australian Barbell Company, Mordialloc, VIC, Australia) and Garage gym brand bumper weight plates on a rubber matted area. Standard deadlift protocol required candidates to stand inside the hexagonal bar with feet placed hip-width apart. When ready, the candidate would squat down to grasp the bar while keeping heel contact with the floor and the head in a neutral position. The lift started with arms fully extended and progressed to an upward lift of the bar while extending the knees and hips. Once standing the candidate placed the bar back on the ground in a smooth, controlled manner (1 repetition). Candidates completed the deadlift on their own and used the Jim Wendler formula to convert their 3 RM to a 1 RM estimate.

Grip strength: Grip strength of both dominant and non-dominant hands were measured using a handgrip dynamometer (Takei Scientific Instruments, Niigata City, Japan). The task was standardized by adjusting the dynamometer so that the base of handgrip rested on the first metacarpal and the handle was in contact with the middle aspect of the four fingers. The candidates were required to squeeze the dynamometer with maximal isometric effort. Candidates were permitted one attempt before the value was recorded to the nearest kilogram.

Loaded pull-ups: On the barrier test day, the loaded pull-up was performed using a 17 kg plate carrier vest. The pull-ups were conducted using an under-grasp grip (back of hands facing away from officer) with a grip width wider than shoulder width to allow for a 90 degree angle when upper arms were parallel to the ground. Candidates were instructed to flex their knees to 90 degrees with ankles crossed behind the body. The 17 kg weighted plate carrier vest was worn over the shoulders with weight distributed on front and back of the upper body. To complete a successful repetition, the candidate could not swing the legs during the pull-up movement and the chin was required to raise above the level of the bar. Pull-ups were repeated until fatigue and the maximum number successfully performed was recorded.

7 stage sit-up: The 7 stage sit-up test is used to measure the relative strength of the abdominal muscles and was administered using a standardized approach as per Dortkamp [20]. In brief, the candidates were required to start in a sit-up position, lying on their back with hands by their sides and knees bent to 90 degrees. Each candidate was asked to perform a controlled movement, as outlined in the protocol, until reaching the final stage if able. With each sequential stage the requirements increase in difficulty, with the final stage requiring the candidate to perform a sit-up with a 5 kg weight behind their head. The final stage that was successfully completed was recorded as the candidate’s final score. Candidates were required to achieve a minimum score of 3 to be successful.

Timed push-ups: The push-up assessment for muscular endurance involved completing as many good-quality repetitions as able in 60 s. Candidates began in a prone position with arms fully extended, only hands and feet were in contact with the ground. Once instructed to begin, the candidates were asked to bend at the elbows and lower their body to the floor. Candidates lowered to the floor until their upper arms were parallel with the ground and then extended their elbows until returning to the starting position (1 repetition). To be considered successful, candidates were required to produce smooth and controlled movements. The final full repetition that was successfully completed was recorded as the candidate’s final score. Candidates were required to achieve a score greater than 40 to be successful.

The 20 m progressive shuttle run test (PSRT): The PSRT is a multi-stage fitness test used to measure the candidates maximal aerobic power, the test was administered following standard procedure as described by Robinson et al. [19]. In brief, the test requires candidates to complete 20 m shuttle runs before the next successive beep (administered by audio compact disc from the Australian Sports Commission). Shuttle runs are performed continuously, with successive increases in speed after each level until voluntary exhaustion is reached. If the candidate achieves two consecutive failed attempts their score is recorded, and the candidate is withdrawn from the test. During the assessment, heart rates (HR) were monitored using the Team Polar HR system, to ensure near maximal/maximal efforts. Scores were recorded in Level and Stage (e.g., level 10, stage 6) and converted to the number of shuttles performed in total.

1.2 km Run: Candidates were required to complete a distance of 1.2 km (0.74 miles) on foot in shorts, t-shirts and shoes of their choosing. The distance was identified with markers and candidates were required to complete the distance as quickly as possible. Results were recorded in minutes and seconds using a handheld stopwatch. The use of stopwatches to record run times in these populations has been reported in the literature and is common practice due to the venue, size of testing group and ease [1]. Furthermore, stopwatch use as a viable alternative to other forms of electronic timing for the accurate and reliable collection of group data is likewise acknowledged in the literature [21].

Occupational-specific physical assessment (OSPA): On the first day of the selection course (two weeks after the above testing), candidates conducted the OPSA following a 15 min formal warm up. As per dedicated and pre-established protocols, the assessment was conducted on a dry, non-slip surface wearing standard specialist police attire and loads (‘black role’ mean load weight 28.43 ± 0.54 kg) as required by all candidates. Some variable external load between candidates did exist due to differences in body armour size and personal preferences. The candidates completed initial familiarization run throughs until comfortable with the requirements before a final testing run was completed.

**Figure 1:**
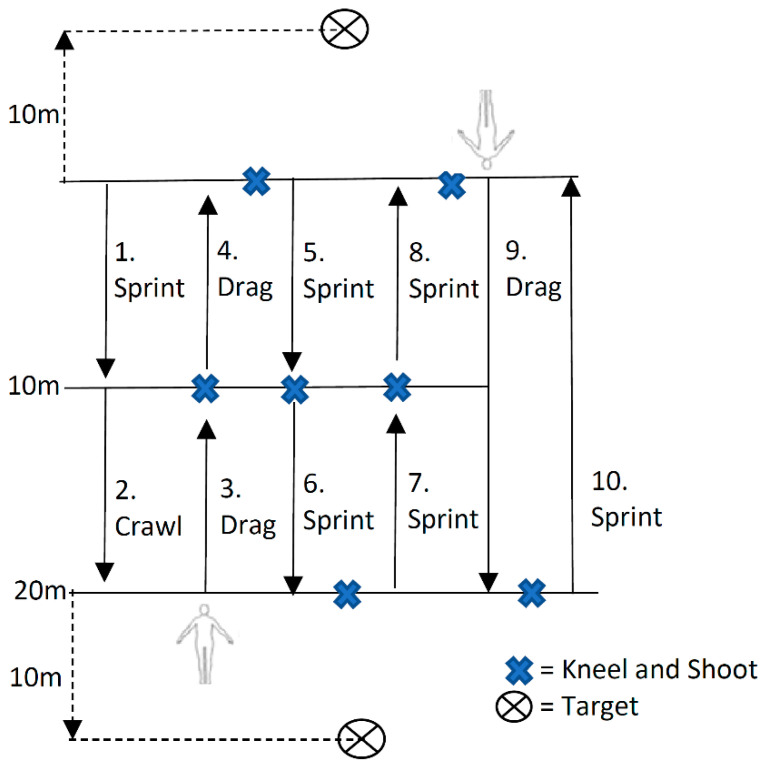
Occupational-specific physical assessment (OSPA) course layout and conduct.

Correct set up of the assessment required six marker cones, a 70 kg mannequin dummy with a 10 kg plate carrier vest secured over the mannequin upper body, handheld stopwatch and recording equipment. Candidates started at base line, facing towards a series of parallel lines identified with marking cones placed at distances of 10 m and 20 m (Figure 1). Once on the base line, candidates were asked to stand with one foot on or behind the starting line with their muzzle raised and aimed at a target in front of them (up to 30 m away). On command, candidates were required to complete a series of timed sequential actions as follows: (1) sprint to the first opposing 10m parallel line; (2) upon arriving at the 10 m mark, candidates were to quickly drop into a prone position and crawl to the next opposing 20 m line; (3) on reaching the 20 m mark opposite the starting line, candidates were required to sling their primary weapon, and raise the upper body of an 80 kg dummy off the ground and quickly drag the dummy backwards until the 10 m line was reached; (4) on reaching the 10 m line, the dummy was to be lowered to the ground, candidates dropped to a kneeling unsupported position, raised the muzzle of their primary weapon and took aim at a target with accuracy confirmed by the assessor when the laser sighting system aligned and was held momentarily on the target (termed sight picture); (5) on achieving a sight picture, the candidate again slung their weapon and picked up the dummy and dragged it a further 10 m backwards, where, on arrival of the starting line, the dummy was lowered and a kneeling firing position was taken; (6) once achieving a sight picture on the target, the candidate stood up and sprinted to the 10 m mark where the same kneeling unsupported firing position was adopted and a sight picture taken; (7) this action was repeated to the 20 m line and was followed by a 180 degree turn (keeping lowered body posture) leading into the same kneeling firing position; (8) on achieving a sight picture on a second target (behind the starting line), the candidate stood up and sprinted back to the 10 m marker and, again, adopted the firing position and sight picture; (9) this was repeated once more, where, on reaching the starting line, the candidate slung their weapon, picked up and dragged the dummy as quickly as possible for the full length of the course (20 m) before again adopting the kneeling firing position (facing towards the starting line); (10) on achieving the sight picture, the candidate stood and sprinted as quickly as possible to the start line to finish the assessment.

If the candidate fell over, they were given up to three minutes recovery time before recommencing the assessment. The overall time to complete the OSPA was measured in minutes and seconds for each candidate. There were no required standards to pass the assessment, but all candidates were required to complete the course as fast as possible and were under the scrutiny of the barrier day testing staff.

### 2.3. Statistical Analysis

Data were provided in a Microsoft Excel spreadsheet and imported for analysis into Statistical Package for the Social Sciences (SPSS v.25.0, SPSS Inc., Chicago, IL, USA). Descriptive statistics (minimum; maximum; mean ± standard deviation (SD)) were calculated for each variable. Following these descriptive analyses, Pearson’s correlations, for anthropometric data and for fitness measures identified in the regression, were performed to determine the strength of relationships. Correlation strength was designated as: an r between 0 and ±0.3 was considered small; between ±0.31 and ±0.49, moderate; between ±0.5 and ±0.69, large; between ±0.7 and ±0.89, very large; and between ±0.9 and ±1 was considered near perfect [1,22]. A stepwise linear regression was conducted to determine the influence of measured fitness parameters on OSPA performance. To minimize multi-collinearity among variables, variables that had a very large (0.7+) correlation with another variable were not included in the regression. An alpha level of *p* < 0.05 was set a priori. A power analysis indicated that the available sample size of 18 personnel would provide 80% power to detect a moderate correlation of 0.6 between predictor variables and the outcome of interest, with an alpha level of 0.05.

## 3. Results

Descriptive data for anthropometric, fitness, and performance (OPSA) measures for all 18 candidates are shown in Table 1. The Pearson’s correlations between anthropometric measures and the OSPA revealed no significant relationships between height, weight, BMI and LBMI and OSPA performance (Table 2).

A moderate negative correlation was found between 1 RM shoulder press and OPSA (r = −0.531, *p* = 0.23), indicating that as shoulder press strength went up, OSPA performance times went down (Table 3). No other significant correlations were found between fitness measures and OPSA performance. The Pearson’s correlations between fitness variables identified grip strength in the non-dominant hand, the Illinois agility test and the 1 RM hex deadlift as having very strong relationships with other variables and as such these variables were excluded from the regression (full table of correlations is presented in Appendix A Table A1). The stepwise linear regression data featured both the 1 RM military shoulder press and grip strength of the non-dominant hand as the most significant predictor of performance on the OSPA, accounting for 60.2% of the variance and 54.9% of the variance explained by the independent variables impacting on the dependent variable alone (*p* = 0.001) (See Table 4). A separate model, exclusively using the 1 RM military shoulder press predicted OSPA performance, accounting for 28.2% of the variance in performance and 23.7% (*p* = 0.023) of the variance explained by the independent variable impacting on the dependent variable alone. Although both the 1 RM shoulder press and grip strength were featured in the regression, the only significant independent factor was the shoulder press.

## 4. Discussion

The purpose of this study was to establish the relationships between select measures of physical fitness and performance on an OSPA. The outcomes of this study suggest that both shoulder and grip strength were large and significant predictors of OSPA performance and had an even stronger predictive relationship when considered together. Interestingly, several variables commonly used in law enforcement organizations, e.g., push-ups, sit-ups and grip strength [1,23], were not related to performance on the OPSA (see Table 1). These results signify the importance of upper body strength in a multiple-event specialist tactical response police task. Additionally, developing and maintaining upper body muscular strength may positively influence loaded complex physical skills undertaken while operating in the field.

The military shoulder press and grip strength assessment were both employed as maximal strength tests in this study. A potential reason for their predictive ability may be the need for greater shoulder and grip strength when performing critical occupational tasks seen in the OSPA such as crawling, quickly pushing one’s self up from the ground, controlling a firearm and grasping and dragging a dummy with maximal or near maximal effort. With limited research available regarding a task assessment equivalent to the OSPA, making a direct comparison is difficult. However, similar to the findings of this study, past research has shown high correlations between upper body strength and more complex job requirements. Robinson et al. [19], found a moderate-to-large and significant correlation between both 1 RM pull-ups (*r* = −0.512 to −0.607, *p* < 0.01) and 1 RM bench press (*r* = −0.295 to −0.360, *p* < 0.05) and performance on a 5 km pack march (25 kg external load) in specialist police. Lockie et al. [1] demonstrated that scores on both pull-ups and push-ups in law enforcement recruits highly correlated with task performance on both a 99 yard obstacle course and chain link fence climb, while pull-ups alone predicted performance on a solid wall climb. Similar to the aforementioned studies, it has been widely acknowledged that both recruits [24] and law enforcement officers [25] who score higher on fitness measures tend to perform better on job-specific tasks. The characteristics of specialist tactical response personnel might explain these correlations, as these officers frequently perform specialist tasks such as victim rescues, forceful entries, grappling, handling firearms and ballistic shields and apprehending suspects [3,23]. Of note, a variety of these occupational tasks are performed on the OSPA, such as a simulated victim drag and handling a firearm. The use of upper body strength and endurance is likely beneficial for many of these tasks, which highlights why there may be strong relationships seen between these measures and performance on a multiple task event.

Similar to the current study, past research has also explored the relationship between grip strength and performance on occupational tasks. Noting that it was only included in the second model, the correlation between grip strength and performance on the OSPA in the current study contrasted with that of other studies. Comparing these findings to Michaelides et al. [26], the influence of grip strength on a dummy drag was highlighted in a cohort of 90 firefighters with similar descriptive data for anthropometric and grip strength measures. Of importance was the moderate and significant relationship established between a rescue mannequin drag (82 kg dummy) and grip strength (*r* = −0.41, *p* < 0.01). In a comparable cohort of firefighters, Nazari et al. [27] similarly found a moderate correlation between dominant grip strength and performance on a hose drag (*r* = −0.25, *p* < 0.05). Although less heavy, firefighters performed a similar task while wearing full personal protective equipment (22.7 kg), a similar load to the cohort in this study. Of note, however, the drag task in this paper was part of a multi-task circuit and occurred several times. As such, grip endurance may be a more important factor. Furthermore, research by Muirhead et al. [28] has shown that grip strength can range from negatively (*r* = −0.367) to positively (*r* = 0.040) correlating with police officer marksmanship task (aim was the highest possible score) depending on the nature of the shooting task. As such, the nature of the marksmanship component in the OPSA may have influenced the grip strength requirement to align and hold a sight picture on the target with their weapon and, as correct sight pictures were a requirement before progressing in the OPSA, may have influenced outcome times.

It is interesting to note that no other fitness measures significantly correlated with OSPA performance. Given how strong the correlation was between the two upper limb strength measures and the OSPA, a likely possibility to describe the weaker correlation seen amongst upper limb and trunk endurance, lower limb and aerobic measures could be the existence of a ceiling effect. The ceiling effect may likely be attributable to the already high fitness levels observed in specialist personnel [7,11], as such they already possess adequate lower limb strength, muscular endurance and cardiovascular endurance to successfully perform the given task at a high level; a supposition supported by Tomes et al., in a study of police recruits [29]. The average PSRT score of 11.45 would equate to a relative VO2 max of 51.7 mL/kg/min, which would place them in the 90th percentile for their age group [30]. A critical review by Maupin et al. [11] found higher relative VO2 max and muscular endurance scores in elite tactical units (ETUs) when compared to general officers and populations but found no significant difference between populations in terms of muscular strength, power and sprint speed. This supports the potential of a ceiling effect in the current study, as the candidates should be expected to already possess superior levels of muscular and cardiovascular endurance, thus no significant correlation was found. Maupin et al. [11] additionally stated that, although no collective significant differences were seen in strength among populations, when looking exclusively at the 1 RM bench press, ETUs did rank higher than the general population. This may help explain the reason for a correlation with the shoulder press but not bench press. Although not measured in the current study, it would be worthwhile to determine the effects of power and speed on the OSPA or similar occupational tasks given the findings of the aforementioned study. This highlights the need for continued research in this area.

Another finding of interest was the lack of a significant correlation between BMI, LBMI and OSPA performance. Although not a key focus in the current study, LBMI did have a large and significant correlation with lower limb strength measures (*r* = 0.647, *p* = 0.004), whereas BMI more strongly correlated with upper limb strength measures (*r* = 0.632, *p* = 0.005). As this is the first study to utilise LBMI, there is a lack of suitable studies to compare the findings. This implies that there is further room for research in this area and how it may impact both fitness measures and occupational tasks performed by officers.

The findings of this study highlight the benefits for law enforcement agencies to create and utilise more task specific conditioning assessments and programs, such as the OSPA. This is supported by both Dawes et al. [31] and Irving et al. [9], who stress the importance of aligning physical conditioning with the specific demands of relevant occupational tasks and environments. The current study has aided in the understanding of how fitness measures relate to specialist police task performance and thus will help create more appropriate conditioning plans to increase mission success. However, there is opportunity for further investigation regarding the relationship between physical assessment measures and task performance, as it may vary notably between different operational tasks and environments (e.g., load carried, high-risk warrant searches vs. reconnaissance mission, urban vs. rural) [9].

There are existing limitations in this study which should be acknowledged. First, the small sample size (*n* = 18). However, seeing as other studies have utilised smaller sample sizes, eleven [7] and six [32] in this specialized population, the current study did comprise a relatively large sample size for this exclusive population. Other investigations in this field have used sizes of between 12 and 18 [14,33,34], which can be the entire unit in some situations. While this may be a criticism, the sample size was the entire group of candidates who attended the selection course. A second potential limitation is the use of only male candidates. As females may have different physiological characteristics and capabilities, the current study may not be representative of the entire population being investigated noting that this population is almost exclusively comprised of male personnel. Further research should be conducted to support the current findings in female participants. Finally, the marksmanship task used alignment of trigger pressured laser designators with the target as an indication of successful target engagement. Given that no live ammunition was used and no consequence for a target miss existed, the actual speed of target engagement and hence overall times may have been quicker than if live ammunition was used. Future research should explore differences in the use of laser designator, simunition and live ammunition in target engagements to determine if these different approaches would influence OPSA times.

## 5. Conclusions

The findings in this study suggest the importance of upper limb muscular strength on the performance of an OPSA in specialist police candidates. It appears that both shoulder and grip strength may play a crucial role in determining performance on a series of job-specific tasks including a dummy drag, handling a weapon and other loaded dynamic movements and may become of increasing importance if, and when, the lower body and aerobic capacity have reached a certain threshold. By implementing and tracking these select fitness measures, the ability of these officers to successfully perform occupational tasks may be enhanced.

## Figures and Tables

**Table 1:** Descriptive data for demographic, anthropometric, physical fitness and occupational task performance measures in specialist police candidates.

Variable	Min	Max	Mean ± SD
Age (years)	26	42	32.1 ± 5.04
Height (cm)	173	193	183.72 ± 5.79
Weight (kg)	71	110	89.44 ± 8.56
BMI (kg/m^−2^)	23.18	29.53	26.45 ± 1.58
LBMI (kg with load/m^−2^)	30.65	35.97	33.60 ± 1.61
Illinois agility (sec)	15.44	16.91	15.93 ± 0.52
1 RM bench press (kg)	97.5	130	114.31 ± 8.13
1 RM back squat (kg)	100	160	132.72 ± 16.01
1 RM military shoulder press (kg)	47.5	80	65.64 ± 9.07 *
1 RM hex deadlift (kg)	134	185	166.39 ± 15.91
Grip strength: Dominant (kg)	50.3	72.9	61.95 ± 7.00
Grip strength: Non-dominant (kg)	47.2	66.7	60.17 ± 5.40
Loaded pull-ups + 17 kg plate carrier (reps)	3	10	6.39 ± 2.17
7 stage sit-up (reps)	5	7	6.78 ± 0.55
Push-ups: 60 sec (reps)	49	77	60.28 ± 7.63
PSRT (level)	10.1	13.1	11.48 ± 1.06
1.2 km run (min)	4.01	5.14	4.28 ± 0.26
OSPA (min)	01:36.8	02:07.5	01:51.73 ± 00:09.21
OSPA load weight (kg)	27.45	29.5	28.43 ± 0.54

Key: BMI = body mass index; LBMI = loaded body mass index; OSPA = occupational-specific physical assessment. * Significantly correlated with OPSA performance, *p* = 0.023.

**Table 2:** Pearson’s correlations between demographic and anthropometric measures and the OSPA.

Assessment	Relationship	Age	Height	Body Weight	BMI	LBMI
OPSA	Correlation	0.07	−0.12	−0.18	0.12	0.17
	Sig.	0.79	0.64	0.48	0.66	0.51

Key: BMI = body mass index; LBMI = loaded body mass index; OSPA = occupational-specific physical assessment.

**Table 3:** Pearson’s correlations between fitness measures and the OSPA.

Relationship	Illinois Agility	1 RM Bench Press	1 RM Back Squat	1 RM Military Shoulder Press	1 RM Hex Deadlift	Grip Strength (D)	Grip Strength (ND)	Loaded Pull-Ups	7 Stage Sit-Up	Push-Ups	PSRT	1.2 km Run
*r*	0.229	−0.204	−0.227	−0.531 *	−0.304	0.408	0.354	−0.065	−0.274	0.064	−0.215	0.332
Sig.	0.361	0.417	0.366	0.023	0.220	0.093	0.150	0.797	0.271	0.802	0.391	0.178

Key: * Significant *p* < 0.05; 1 RM = 1 repetition maximum; D = dominant; ND = non-dominant.

**Table 4:** Unstandardized (B) and standardized (β) regression coefficients for each predictor variable.

Variable	B [95% CI]	β	*p*
Model 1 (*r^2^* = 0.282)			
1 RM Shoulder Press	−0.54 [−1.00, −0.83]	−0.53	0.023
Model 2 (*r^2^* = 0.602)			
1 RM Shoulder Press	−0.70 [−1.07, −0.33]	−0.68	0.001
Grip Strength (dominant)	0.78 [0.229, 1.25]	0.59	0.003

## Appendix A

**Table A1:** Correlations between fitness measures.

Assessment	Grip Strength: Dominant (kg)	Grip Strength: Non-Dominant (kg)	7 Stage Sit-Up (reps)	Push-Ups (reps)	PSRT (level)	1.2 km Run (min)	Illinois Agility (sec)	Loaded Pull-Ups + 17 kg Plate Carrier (reps)	1 RM Bench Press (kg)	1 RM Back Squat (kg)	1 RM Military Shoulder Press (kg)	1 RM Hex Deadlift (kg)
Grip strength: Dominant (kg)	1											
Grip strength: Non-dominant (kg)	0.856 **	1										
7 stage sit-up (reps)	−0.043	0.023	1									
Push-ups (reps)	−0.014	0.104	0.311	1								
PSRT (level)	−0.131	−0.009	0.557 *	0.326	1							
1.2 km run (min)	0.283	0.019	−0.551 *	−0.432	−0.619 **	1						
Illinois agility (sec)	0.355	0.211	−0.781 **	−0.335	−0.792 **	0.788 **	1					
Loaded pull-ups + 17 kg plate carrier (reps)	−0.041	0.060	0.373	0.625 **	0.138	−0.367	−0.337	1				
1 RM bench press (kg)	0.115	0.156	−0.202	0.305	−0.108	−0.114	0.264	−0.084	1			
1 RM back squat (kg)	0.154	0.453	0.240	0.394	0.290	−0.409	−0.124	0.044	0.553 *	1		
1 RM military shoulder press (kg)	0.260	0.350	0.119	0.276	0.165	−0.360	0.005	0.110	0.562 *	0.493 *	1	
1 RM hex deadlift (kg)	0.303	0.503 *	−0.057	0.162	0.203	−0.323	0.073	−0.049	0.623 **	0.759 **	0.756 **	1

**. Correlation is significant at the 0.01 level (two tailed) *. Correlation is significant at the 0.05 level (two tailed). 1 RM = 1 repetition maximum; PSRT = progressive shuttle run test.
